# The Chemopreventive Effects of Protandim: Modulation of p53 Mitochondrial Translocation and Apoptosis during Skin Carcinogenesis

**DOI:** 10.1371/journal.pone.0011902

**Published:** 2010-07-30

**Authors:** Delira Robbins, Xin Gu, Runhua Shi, Jianfeng Liu, Fei Wang, Jacqulyne Ponville, Joe M. McCord, Yunfeng Zhao

**Affiliations:** 1 Department of Pharmacology, Toxicology and Neuroscience, Louisiana State University Health Sciences Center, Shreveport, Louisiana, United States of America; 2 Department of Pathology, Louisiana State University Health Sciences Center, Shreveport, Louisiana, United States of America; 3 College of Life Science, Jilin University, Changchun, Jilin Province, China; 4 Department of Chemistry, Nicholls State University, Thibodaux, Louisiana, United States of America; 5 Department of Medicine, University of Colorado at Denver and Health Sciences Center, Aurora, Colorado, United States of America; 6 Feist-Weiller Cancer Center, Louisiana State University Health Sciences Center, Shreveport, Louisiana, United States of America; Institut Européen de Chimie et Biologie, France

## Abstract

Protandim, a well defined dietary combination of 5 well-established medicinal plants, is known to induce endogenous antioxidant enzymes, such as manganese superoxide dismutase (MnSOD). Our previous studies have shown through the induction of various antioxidant enzymes, products of oxidative damage can be decreased. In addition, we have shown that tumor multiplicity and incidence can be decreased through the dietary administration of Protandim in the two-stage skin carcinogenesis mouse model. It has been demonstrated that cell proliferation is accommodated by cell death during DMBA/TPA treatment in the two-stage skin carcinogenesis model. Therefore, we investigated the effects of the Protandim diet on apoptosis; and proposed a novel mechanism of chemoprevention utilized by the Protandim dietary combination. Interestingly, Protandim suppressed DMBA/TPA induced cutaneous apoptosis. Recently, more attention has been focused on transcription-independent mechanisms of the tumor suppressor, p53, that mediate apoptosis. It is known that cytoplasmic p53 rapidly translocates to the mitochondria in response to pro-apoptotic stress. Our results showed that Protandim suppressed the mitochondrial translocation of p53 and mitochondrial outer membrane proteins such as Bax. We examined the levels of p53 and MnSOD expression/activity in murine skin JB6 promotion sensitive (P+) and promotion-resistant (P-) epidermal cells. Interestingly, p53 was induced only in P+ cells, not P- cells; whereas MnSOD is highly expressed in P- cells when compared to P+ cells. In addition, wild-type p53 was transfected into JB6 P- cells. We found that the introduction of wild-type p53 promoted transformation in JB6 P- cells. Our results suggest that suppression of p53 and induction of MnSOD may play an important role in the tumor suppressive activity of Protandim.

## Introduction

Apoptosis is an intricate pathway triggered by various sources, such as genotoxic stress, DNA damage, cytotoxicity and irradiation. It involves both transcription-dependent, as well as, post translational processes. Apoptosis is deeply involved in the early stage of skin carcinogenesis [1]. With carcinogenesis being a multifactorial disease that involves cell proliferation, inflammation and oxidative stress-mediated signal transduction, the two-stage skin carcinogenesis model uses a chemical-induced carcinogenesis approach to study the biochemical and histological changes that occur in various stages of tumorigenesis. Initially, a subcarcinogenic dose of 12-dimethylbenz[a]anthracene [DMBA] is applied to initiate DNA damage resulting in the formation of Ras-mutated skin cells. During the initial stages of skin carcinogenesis, a tumor promoting agent, such as the phorbol ester, 12-*O-*tetradecanoylphorbol-13-acetate, [TPA] is repeatedly applied to the skin to promote the clonal expansion of Ras- mutated cells. During DMBA/TPA treatment, both tumor suppressor gene and oncogene activation occur simultaneously leading to downstream oxidative stress propagation. Consistent with that, TPA is known to induce oxidant formation and subsequent damage to macromolecules [2]. Consequently, the cellular response has been shown to increase skin epidermal hyperplasia and inflammation. The tumor suppressor p53 is also activated during this process. Interestingly, there's also an increase in p53 mitochondrial translocation. Other than a well-known skin tumor promoter, low concentrations of TPA have been shown to induce apoptotic cell death either alone or in combination with anti-cancer drugs in human pancreas cancer cells [3], and human prostate cancer cells [4], [5]. In addition, it has been further demonstrated that apoptosis precedes cell proliferation [1] which clearly supports the notion that cell death is another key contributing event during cancer development. Oxidative stress has been recognized to play a contributing role in cancer development. However, studies have shown that oxidative stress is a mediator of apoptosis. Cellular homeostasis relies on the balance between pro-oxidants and antioxidants. However, processes such as oxidative stress shift this homeostatic balance towards increased pro-oxidant formation. As result, various morphological and biochemical modifications occur that initiate both transcription dependent and post-translational processes of apoptosis. Apoptosis can be characterized by various morphological changes such as DNA fragmentation, cell shrinkage and chromatin condensation. Nevertheless, the activation of the tumor suppressor p53 remains an extensively studied pathway in the field of programmed cell death. p53 is activated early during carcinogenesis and contributes to the propagation of oxidative stress. It has been demonstrated that p53-mediated apoptosis is preceded by activation of various oxidoreductases and reactive oxygen species [ROS] generation prior to mitochondrial perturbation [6]. A fraction of p53 is localized in mitochondria at the onset of p53-dependent apoptosis preceding changes in mitochondrial membrane potential, cytochrome *c* release and caspase activation [7]. Consistent with that, previous studies suggest that mitochondrial p53 physically interacts with manganese superoxide dismutase [MnSOD], leading to inactivation of its enzymatic activity [1]. Interestingly, the reduction of antioxidant activity contributes to oxidative stress propagation which leads to downstream cancer development. Similar results have been observed in UV-induced skin carcinogenesis mouse models [8]. As aforementioned, DMBA/TPA treatment also leads to oncogene activation. It has been demonstrated that the Ras/Rac/NADPH oxidase/p53/apoptosis circuitry may potentially exist in Ras-mutated skin cells. Nevertheless, considerable attention is being focused on the link between p53-induced apoptosis, oxidative stress propagation and mitochondria. Thus, p53 may mediate apoptosis by mechanisms that are both transcriptionally dependent and independent; and the generation of oxidative stress may serve as an important mechanism during carcinogenesis. This poses the question: Can apoptosis be modulated by regulators of oxidative stress? MnSOD is a nuclear encoded primary antioxidant that resides in the mitochondria. Previous studies have demonstrated that overexpression of MnSOD can reduce both tumor incidence and multiplicity in both *in vitro* and *in vivo.* It is known that various dietary components can induce endogenous antioxidant enzymes. We have demonstrated this same paradigm with the use of Protandim, a dietary combination of five extensively studied medicinal plants, given via dietary administration [9]. One capsule of Protandim consists of the following ingredients: *B. monnieri* (45% bacosides), 150 mg, *S. marianum* (70–80% silymarin), 225 mg; *W. somnifera* (1.5% withanolides), 150 mg; *C. sinensis* (98% polyphenols and 45% (-)-epigallocatechin-3-gallate), 75 mg; and *C. longa* (95% curcumin), 75 mg [10]. All of the ingredients of Protandim have individually shown cytoprotective activity in mitigating oxidative stress in both *in* vivo and *in* vitro studies [11]–[16]. Within three-weeks, the Protandim diet was able to significantly induce endogenous antioxidant enzymes such as catalase, MnSOD and copper/zinc superoxide dismutase [Cu/ZnSOD] *in vivo* without signs of overt toxicity. As a result, the Protandim diet exhibited its anti-carcinogenic activity by reducing tumor incidence and multiplicity via modulating oxidative stress through the induction of endogenous antioxidant enzymes [9]. Therefore, further mechanistic insight is needed to determine how modulation of antioxidant expression/activity via Protandim affects p53-mediated mitochondrial functions. In this study, we investigated the effects of Protandim on cutaneous apoptosis, p53 mitochondrial translocation and compared the p53 and MnSOD status between promotable and non-promotable skin epidermal cells.

## Materials and Methods

### 2.1 Cell line, reagents and treatment

Murine skin epidermal JB6 response variants: stably responsive JB6 (P+, CL41) and nonresponsive JB6 (P-, CL30-7b) to tumor promoter-induced transformation were purchased from American Type Culture Collection (ATCC, Rockville, MD). These cells were cultured and maintained as previously described [17]. The cells were grown in EMEM medium supplemented with 4% fetal bovine serum, 2 mM of L-glutamine, 50 µg/ml penicillin and 50 µg/ml streptomycin. TPA (Sigma) was prepared as a 1 mM stock solution in dimethylsulfoxide (DMSO). The TPA stock solution was diluted directly in the cell culture medium, with the resulting concentration being 100 nM.

### 2.2 Anchorage-independent growth assay in soft agar

Soft agar transformation assays were carried out in six-well plates for the experiment. The bottom of each well was coated with 3.5 ml of 0.5% agar in EMEM (10% FBS). A total of 100,000 JB6 cells were suspended in 0.75 ml of 0.33% agar in EMEM (10% FBS), layered on top and incubated for 7 days. To detect the tumor suppressive effect of Protandim in JB6 P+ cells, both layers of agar were supplemented with TPA (5 nM), Protandim extract + TPA, Protandim extract (final dilution: 1 µl/ml) alone, or ethanol (EtOH; vehicle control). The colonies formed were counted via Neutral Red staining. The transformation response was expressed as the number of colonies formed per 100,000 cells per well (the results were shown in Table S1). To detect how p53 affects cell transformation in non-promotable JB6 (P-) cells, cells were seeded in 6-well plates and incubated for 24 h. FuGENE HD reagent (Roche Applied Science) was used to transfect 5 µg HA tagged p53- (wild-type) or GFP-pcDNA3 vectors into JB6 P- cells during a 48 h incubation period. The GFP-conjugated-pcDNA3.1 vector was used to monitor the transfection efficiency and served as a control. Cells were then detached and subjected to the soft agar assay. The transformation response was expressed as the number of colonies formed per 100,000 cells per well.

### 2.3 Detection of cutaneous apoptosis during early stage skin carcinogenesis

Skin tissues were fixed in 4% formaldehyde and processed for histopathology. Apoptotic cells were counted using light microscopy. Ultrastructural features were used to identify apoptosis, such as cell shrinkage, chromatin condensation, formation of cytoplasmic blebs and apoptotic bodies. Conventional electron microscopy of mouse skin tissues was used to examine and photograph apoptotic and mitotic cells using a Hitachi H-600 electron microscope. Dr. Xin Gu, a certified pathologist performed the pathological examination to confirm the morphological characteristics of the cutaneous apoptotic cells.

### 2.4 Dietary prevention of mouse skin carcinogenesis

The two-stage skin carcinogenesis study and dietary administration of Protandim were performed as previously described [9]. Non-tumor tissues were carefully collected for biochemical and histological studies.

### 2.5 Isolation of mitochondrial fraction from skin cells

Skin epidermal cells were stripped and collected as previously described [18]. Cells were then suspended in 2 ml of mitochondria isolation buffer [0.225 M mannitol, 0.075 M sucrose, 1 mM EGTA (pH adjusted to 7.4 with 0.5 M Tris)] in a 10-ml Wheaton homogenizer tube and carefully homogenized three times with 30 s strokes using scale 2 on ice. The cellular debris was removed by centrifugation at 2,500 rpm (∼600 g) twice for 5 min. The supernatant was filtered through a nylon screen cloth (Small Parts, Inc., Miami Lakes, FL) and then centrifuged at 10,000 rpm (∼9,000 g) for 10 min. Supernatant was kept and designated as supernatant fraction. The pellet was washed by adding 0.5 ml of mitochondria isolation buffer and centrifuging at 10,000 rpm for 5 min. This washing was repeated twice. The mitochondrial pellet was resuspended in 50–100 µl of mitochondria isolation buffer containing the protease inhibitor cocktail (Research Products International Corp.; Mount Prospect, IL). This fraction was labeled as the mitochondria fraction and kept at −80°C. The purity of the mitochondrial fractions has been confirmed by trace contamination of a nuclear marker, proliferating cell nuclear antigen, [PCNA].

### 2.6 Preparation of total cell lysate of JB6 cells

JB6 cells were collected by centrifugation and resuspended in RIPA Buffer (50 mM Tris, 150 mM NaCl, 0.1% SDS, 0.5% Na. Deoxycholate, and 1% Triton ×100) supplemented with the protease inhibitor cocktail (5 µg/ml each of pepstatin, leupeptin, and aprotinin) for 10 s (Sonic Dismembrator Model 100, scale 2, Fisher Scientific), sonicated, incubated on ice, and centrifuged.

### 2.7 Western blot analysis

Mitochondrial fractions prepared from the mouse skin tissues were used to detect mitochondrial p53 and Bax expression. The whole cell lysate prepared from JB6 cells was used to detect the induction of p53 and Bax expression by TPA. Thirty micrograms of the protein samples were separated on a 10% SDS-PAGE gel and transferred to nitrocellulose membrane. Ponceau staining was used to monitor the uniformity of the transfer. The membrane was blocked in Blotto [5% milk, 10 mm Tris-HCL (pH 8.0), 150 mM NaCl and 0.05% Tween-20] for 1 h at room temperature. Anti-p53 antibody (FL-393, Santa Cruz Biotechnology, Santa Cruz, CA) and anti-Bax antibody (P-19, Santa Cruz Biotechnology, Santa Cruz, CA) were added in 1∶1000 dilutions and the membrane was incubated for 2 h at room temperature. After washing, the membrane was incubated with horseradish peroxidase conjugated secondary antibodies (Santa Cruz) at a 1∶1000 dilution. For loading controls, succinate dehydrogenase subunit B, anti-SDHB (FL280, Santa Cruz) was used as a mitochondrial marker; and glyceraldehyde-3-phosphate dehydrogenase, anti-GAPDH (Santa Cruz) for total cell lysate. The antibody bands were visualized by the enhanced chemiluminescent detection system (ECL, Amersham). Image J 1.38× Software was used for densitometric analysis for protein bands obtained by Western blot analysis, and the ratio of the density of the target protein to the density of the loading control was plotted and analyzed using GraphPad Prism 3 software.

### 2.8 MnSOD activity assay

The nitroblue tetrazolium- bathocuproine sulfonate (NBT-BCS) assay was used to measure the MnSOD activity. Total cell lysate was prepared in 50 mM phosphate buffer. The assay buffer contained xanthine-xanthine oxidase which was responsible for superoxide generation. As a result, NBT was reduced by superoxide to form the blue product formazan. The presence of MnSOD inhibited the NBT reduction. The data was plotted as units per milligram of protein. One unit of activity was defined as the amount of protein needed to inhibit 50% of the NBT reduction rate. NaCN (5 mM) was used to measure MnSOD activity.

### 2.9 Detection of the levels of the reactive oxygen species (ROS) in JB6 cells

JB6 (both P- and P+) cells were seeded into 96 well plates (1×10^5^ cells per well) and incubated overnight. The next day, growth medium was replaced with fresh medium containing the vehicle (0.1% DMSO) or TPA (100 nmol), and cells were incubated for 1 h. Medium was removed and replaced with fresh medium containing 10 µM H_2_DCF-DA (Molecular Probes, Eugene, OR), and cells were incubated for 15 min. DCF fluorescence was detected using a fluorescence plate reader (Synergy HT, BioTek, Winooski, VT; excitation: 485 nm; emission: 528 nm).

### 2.10 Statistical analysis

Student's t-test was used for two-group comparison, and one-way ANOVA followed by Newman-Keuls post-test was used for multi-group comparisons. Data were reported as means ± standard error (S.E.M.). p<0.05 was considered significant. For experiments including both TPA and Protandim treatments, two-way ANOVA was used followed by the Tukey-Kramer method for multiple comparisons. Two-way analysis of variance (ANOVA) was used to assess the effects of TPA and Protandim on the number of apoptotic cells present per 100 cells. Since there was an interaction effect of Protandim and TPA on number of apoptotic cells and the sample size are unequal among the factors, the least square means were estimated and compared among the combinations of these two factors. Tukey-Kramer method was used in the adjustment for multiple comparisons. Statistical software SAS system 9.3 (SAS Inc. Gary, NC) was used for two-way ANOVA data analysis.

## Results

### Protandim suppressed DMBA/TPA induced apoptosis

**Figure 1:**
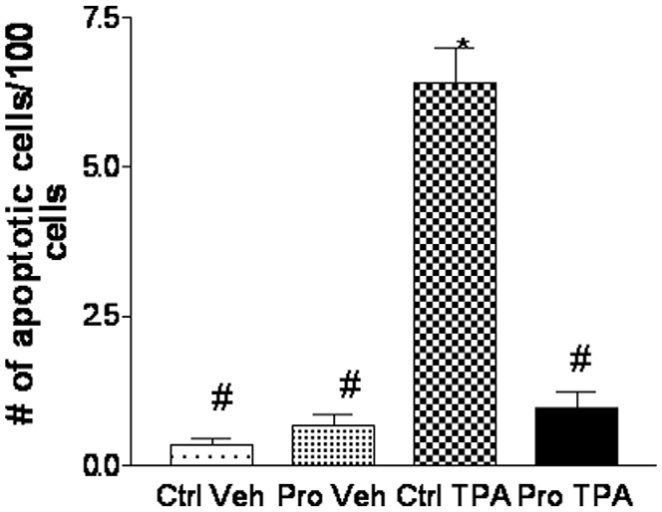
Protandim suppressed DMBA/TPA induced apoptosis. Skin tissues from each treatment group were collected at the end of the skin carcinogenesis study. Skin tissues were fixed and apoptotic cells were counted using light microscopy. The histological examination was confirmed with a pathologist (X.G.) and two-way analysis of variance (ANOVA) was used to assess the effects of TPA and Protandim on the number of apoptotic cells present per 100 cells. Tukey-Kramer method was used in the adjustment for multiple comparisons. Statistical software SAS system 9.3 (SAS Inc. Gary, NC) was used for two-way ANOVA data analysis. *, significantly different from Ctrl Veh: DMSO; #, significantly different from Ctrl/TPA. Ctrl: control basal diet (AIN-76A); Veh: Vehicle control (DMSO); Pro: Protandim-containing diet; TPA: 12-*O*-tetradecanoylphorbol-13-acetate.

Apoptosis is a prominent biological outcome following carcinogen treatment [1], [19]. Histological examination of mouse skin tissues treated with DMBA/TPA also showed frequent apoptosis, in addition to adjacent single cell mitosis. Previous studies have demonstrated that Protandim suppresses cutaneous proliferation and inflammation. Since DMBA/TPA also causes cell death, we investigated the effects of Protandim on cutaneous apoptosis. We found that skin tissues from mice treated with DMBA/TPA exhibited characteristic hyperplasia within the epidermal layer. Within this treatment group, the apoptotic cells were typically close to mitotic cells (an EM picture of an apoptotic cell and a neighboring mitotic cell was shown in Figure S1). The apoptotic keratinocytes resembled shrunken cells with dense staining with an eosinophillic cytoplasm. As summarized in Figure 1, there were approximately 6 apoptotic cells per 100 cells in the DMBA/TPA treatment group. Interestingly, there were less than 1 apoptotic cell per 100 cells in the vehicle control (DMSO) and 1 apoptotic cell per 100 cells in the Protandim diet fed DMBA/TPA group. Overall, the Protandim diet reduced DMBA/TPA-mediated apoptosis.

### Protandim suppressed DMBA/TPA induced p53/Bax mitochondrial translocation

**Figure 2:**
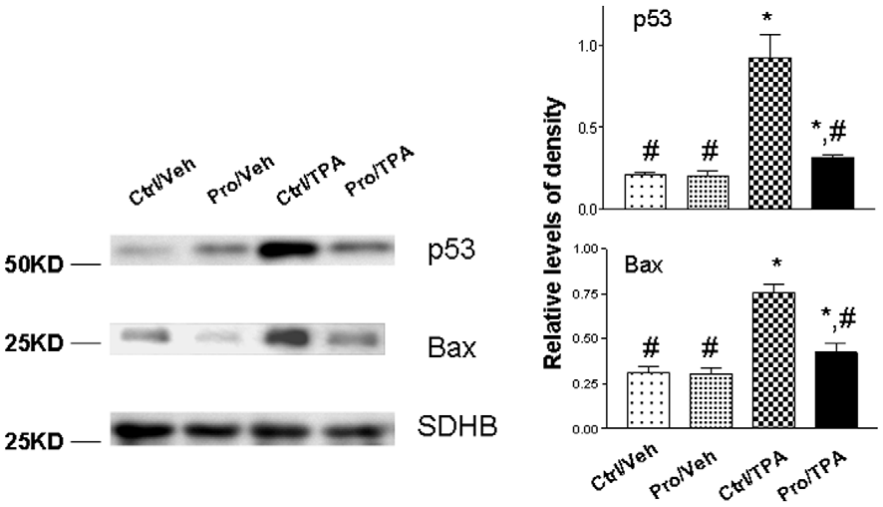
Western blot analysis of p53 and Bax in mitochondrial fraction (left) and examination of apoptosis (right) in mouse skin epidermal tissues. Succinate dehydrogenase subunit B (SDHB) served as the loading control. The levels of p53/Bax were normalized to that of SDHB. Statistical analysis was performed using one-way ANOVA (for multiple group comparison) followed by Newman-Keuls post-test. Ctrl, basal diet; Veh, vehicle control (DMSO); Pro, Protandim. *, p<0.05 when compared to DMBA/TPA group; #, significantly different from Ctrl/TPA.

Previous studies have found that p53 is activated upon TPA treatment; interestingly, p53 also translocates into mitochondria [1]. Since it is known that p53 mitochondrial translocation can mediate apoptosis, we studied the effects of Protandim on p53 mitochondrial translocation. As shown in Figure 2, TPA induced p53 mitochondrial translocation in mice fed the basal diet. However, when given Protandim, via dietary administration, we observed a significant decrease in p53 mitochondrial translocation, suggesting that Protandim suppressed apoptosis by modulating p53-mitochondrial translocation. Bax, as a p53 transcriptional target, is an important player in cellular apoptosis. It has been suggested that p53 interacts with Bax to facilitate mitochondrial translocation [20]. Therefore, we investigated the effects of the Protandim diet on Bax mitochondrial expression. Similarly, we found that TPA induced Bax mitochondrial expression. However, when fed the Protandim diet, we also observed a significant decrease in TPA-mediated Bax mitochondrial expression. This suggests that Protandim, when given via dietary administration, modulates p53 mitochondrial translocation and decreases Bax expression levels in mitochondria.

### TPA induced p53 activation and apoptosis only in promotable, not non-promotable skin epidermal cells

**Figure 3:**
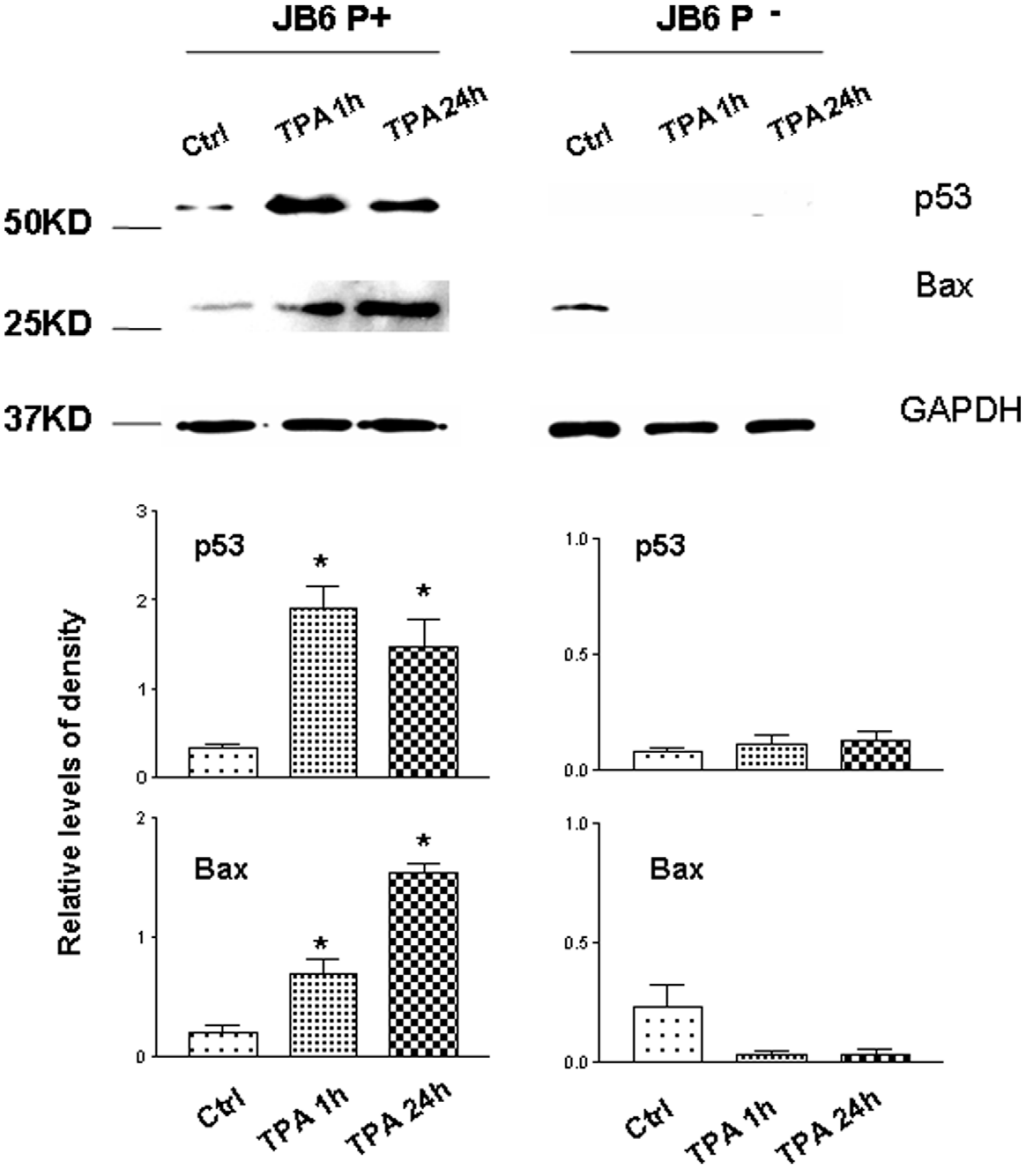
The expression levels of p53 and Bax after TPA (100 nM) treatment in JB6 promotable (P+) and non-promotable (P-) cells. TPA induced p53 activation and apoptosis only in P+ cells, not P- cells. Total cell lysate was used for the assay. The cells were grown in EMEM medium supplemented with 4% fetal bovine serum, 2 mM of L-glutamine, 50 µg/ml penicillin and 50 µg/ml streptomycin. 12-*O*-tetradecanoylphorbol-13-actetate (TPA) was prepared as a 20 nM stock solution in dimethylsulfoxide (DMSO). The TPA stock solution was diluted directly in the cell culture medium, with the resulting concentration being 100 nM. Ctrl: vehicle (0.1% DMSO) treatment for 24 h. *, p<0.05 when compared with the Ctrl group.

Cell death accompanies cell proliferation during skin carcinogenesis. Can early-stage p53 signaling benefit cell growth? We performed our initial studies using clonal variants of the skin epidermal JB6 cell line: tumor promotion sensitive (P+) and -resistant (P-) cells. Herein, JB6 P+ and P- cells were treated with TPA (100 nM) for 1 h and 24 h and compared to vehicle control (DMSO) treated cells to understand the possible involvement of p53 in tumor promotion. As shown in Figure 3, our results indicate that as early as 1 h after TPA treatment, p53 expression was induced in the total cell lysate of JB6 P+ cells. However, this was not seen in JB6 P- cells. This suggests that p53 expression potentially plays a key role in the early induction of tumor promotion. In addition, this global expression of p53 is sustained for 24 h after TPA treatment. Nevertheless, Bax, a pro-apoptotic protein of the Bcl-2 family, was also induced with TPA treatment. Bax, a transcriptional target of p53, is often associated with an increase in apoptosis in targeted cells [21]. To further characterize the role of wild-type p53 in tumor-promotion, promotion-resistant JB6 (P-) cells were transfected with wild-type p53, treated with TPA [5 nM] and analyzed for tumorigenicity using the soft agar assay. Interestingly, wild-type p53 transfected cells formed colonies at a significantly higher level compared to control cells treated in a similar manner (Table 1). Therefore, these results suggest the involvement of p53 signaling in the early stages of tumor promotion.

**Table 1:** Colony Formation in Soft Agar Transformation Assay.

Vector/Treatment	Colonies formed/10^5^ cells
GFP/TPA	13.0±2.65
p53/TPA	33.7±2.96*
GFP/DMSO	0.00±0.00
p53/DMSO	0.30±0.33

*p = 0.0076 compared with the GFP/TPA 5 nM group.

### Non-promotable JB6 cells showed higher MnSOD expression and activity and lower levels of oxidative stress than promotable cells

**Figure 4:**
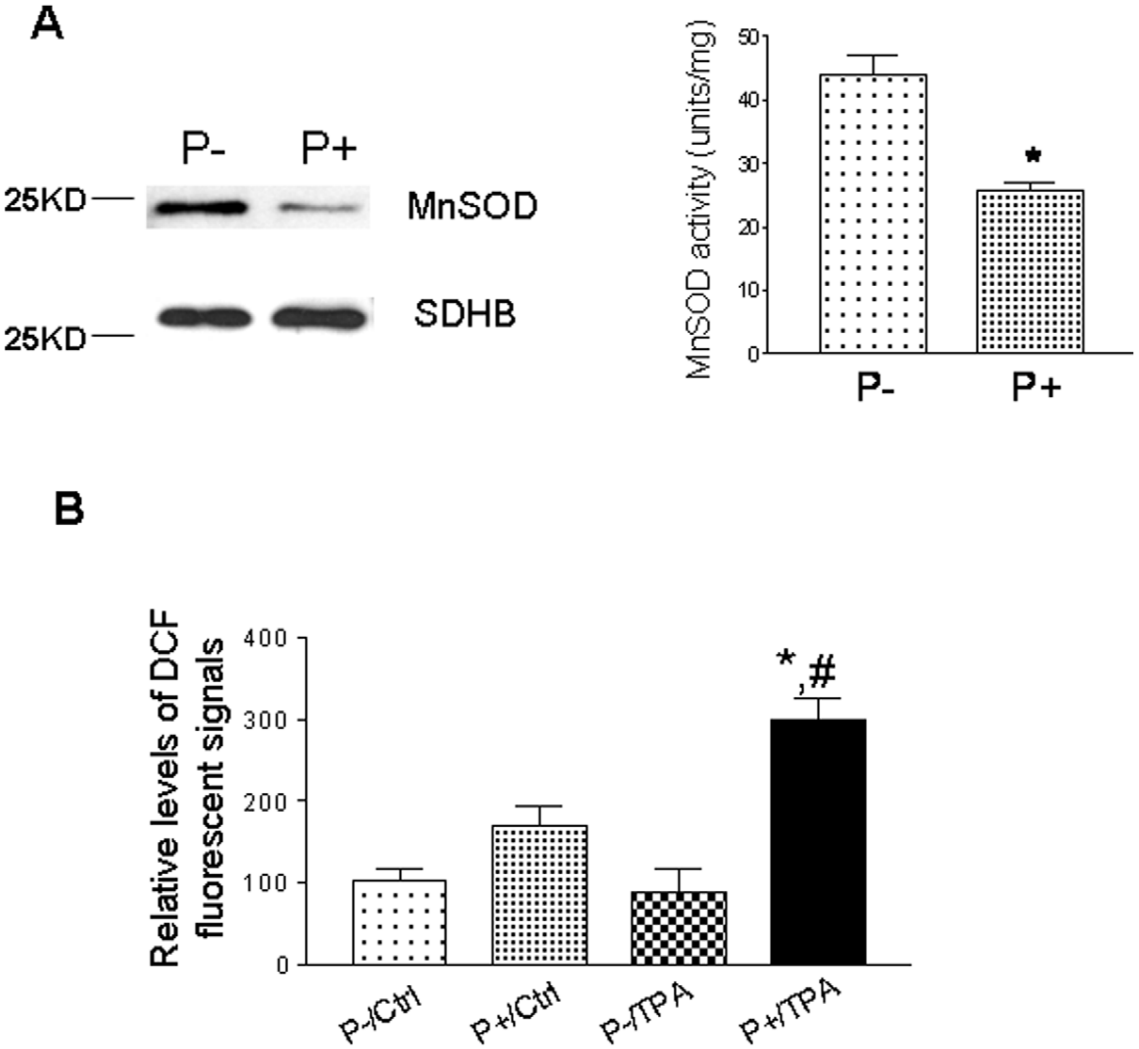
(A). The expression and activity levels of MnSOD between JB6 non-promotable (P-) and promotable (P+) cells. Mitochondrial fractions were used for the experiments. The NBT-BCS SOD inhibition assay was used to measure the MnSOD activity. The presence of MnSOD inhibited the NBT reduction. The data was plotted as percentage inhibition vs. protein concentration. One unit of activity was defined as the amount of protein needed to inhibit 50% of the NBT reduction rate. NaCN (5 mM) was used to measure MnSOD activity. Higher expression/activity levels of MnSOD were observed in JB6 P- cells compared to JB6 P+. SDHB served as the mitochondrial marker and loading control. (B). Detection of ROS levels in JB6 cells using H_2_DCFDA staining. Cells grown in 96-well plates were incubated with TPA or Vehicle (0.1% DMSO) for 1 h following by incubation with 10 µM H_2_DCFDA for 15 min. DCF fluorescence was detected using a fluorescence plate reader (Ex: 485 nm; Em: 528 nm). The fluorescent density was divided by the protein concentration for fair comparison. *, p<0.05 when compared to its control; #, p<0.05 when compared with the TPA group.

MnSOD is a nuclear-encoded primary antioxidant enzyme known to protect the mitochondria from oxidative damage [22]. Previous studies have shown that MnSOD is the only antioxidant enzyme that when overexpressed can suppress tumor incidence and multiplicity. We have also shown that the dietary combination Protandim can induce several endogenous antioxidant enzymes to reduce tumorigenesis in the two-stage skin carcinogenesis mouse model. Protandim induces the antioxidant enzyme, MnSOD, which has been shown to suppress tumorigenesis *in vivo*. In addition, Protandim also suppressed TPA-induced cell transformation of JB6 P+ cells (Table S1). We compared MnSOD *in vitro* levels between JB6 P+ and P- cells. Our studies showed that JB6 P+ cells express lower levels of MnSOD compared to JB6 P- cells (Figure 4A). Molecular cross-talk exists between p53 and MnSOD which leads to reduced MnSOD expression and activity [23], [24]. These results were further confirmed using NBT-BCS SOD inhibition assay. Herein, the results showed significantly lower levels of MnSOD activity in JB6 P+ cells compared to JB6 P- cells. Therefore, tumor promotion sensitive JB6 P+ cells, that express higher levels of p53, have reduced levels of MnSOD expression and activity. Conversely, JB6 P- cells, that possess a tumor promotion resistant phenotype, express lower levels of p53 and higher levels of mitochondrial MnSOD activity and expression. In addition, the levels of oxidative stress in both cell lines were detected using DCF staining with or without TPA treatment. As shown in Figure 4B, without TPA treatment, the ROS levels in JB6 P- cells were approximately 60% of that in JB6 P+ cells. Interestingly, TPA induced increases in ROS levels only in JB6 P+ cells, not in P- cells: with TPA treatment, the ROS levels in JB6 P- were only approximately 30% of that in JB6 P+ cells.

## Discussion

p53-mediated apoptotic signaling still remains an attractive target mechanism in effective chemotherapeutic drug development. For years it has been known that p53 can mediate apoptosis by transcription-dependent mechanisms. However, the cytoplasmic transcription-independent pool of p53 has recently received considerable attention [20]. The tumor suppressor p53 can be activated via DNA damage, hypoxia, oncogene deregulation and oxidative damage. Upon pro-apoptotic stimuli, p53 rapidly translocates to the mitochondria where it physically interacts with Bax, a mitochondrial protein and p53 transcriptional target. Following this interaction, lipid pore formation occurs which allows for p53 mitochondrial entry and the release of apoptotic proteins such as cytochrome *c*; as well as, changes in the mitochondrial membrane potential and caspase activation [25]. Other studies have demonstrated that mice treated with DMBA/TPA exhibited increases in skin epidermal cell proliferation, oxidative stress generation, and apoptosis [19]. In addition, mechanisms that contributed to this dual effect where dependent on AP-1 activation, and p53 expression and localization [19]. Nevertheless, it was found that DMBA/TPA treatment not only significantly increased p53 nuclear accumulation, but there was also a significant increase in p53 mitochondrial expression. In this study, apoptosis is associated with high levels of p53 mitochondrial expression following DMBA/TPA treatment. In addition, signature apoptotic ultrastructural changes, such as cell shrinkage, chromatin condensation and dense nuclear staining are known to occur in mouse skin tissues following DMBA/TPA treatment. Interestingly, we found that DMBA/TPA –mediated apoptotic cells, ultrastructural modifications and p53/Bax mitochondrial translocation were reduced in Protandim-fed mice compared to control mice that were similarly treated. This suggests that modulating ROS generation via the induction of endogenous antioxidant enzymes may regulate p53 mitochondrial translocation. Thus, the endogenous antioxidant enzyme, MnSOD may function as a regulator of apoptosis. However, further studies are needed to clearly elucidate the role of MnSOD in apoptosis alone. Our *in vitro* studies showed that p53/Bax expression could only be induced in tumor promotion sensitive JB6 P+ cells following TPA treatment; however, this was not seen in promotion-resistant JB6 P- cells. To further verify the role of p53 expression in tumor promotion, we transfected promotion-resistant JB6 P- cells with wild-type p53. Interestingly, we found that p53 expression significantly induced colony formation in promotion-resistant JB6 P- cells following TPA treatment. Conversely, when observing MnSOD expression/activity among the JB6 clonal variants, we found that it was the promotion resistant P- cells that expressed higher levels of MnSOD activity and mitochondrial expression. On the other hand, p53 is modulated by TPA-mediated oxidative stress. Our results showed that TPA can also modulate the activation of pro-apoptotic proteins, such as Bax, a downstream p53 transcriptional target. A fraction of p53 is localized to mitochondria at the onset of p53-dependent apoptosis preceding changes in mitochondrial membrane potential, cytochrome *c* release and caspase activation [9]. Recall that once localized in mitochondria, p53 interacts with MnSOD and suppresses its activity. However, this is only one mechanism of p53-mediated MnSOD inactivation. p53 can also bind to the specificity protein -1 (Sp-1) site within the MnSOD promoter region and suppress MnSOD gene expression under both constitutive and TPA-induced conditions [21]. Therefore it's not surprising that promotable cells that possess high levels of p53 activation have low levels of MnSOD expression. Conversely, non-promotable cells that possess low levels of p53 have high levels of MnSOD. Nevertheless, p53 mitochondrial translocation and its physical interaction with MnSOD can also lead to increased ROS generation. Previous studies have shown that mitochondria and mitochondrial-generated ROS contribute to the apoptotic process [26]. However, we have demonstrated that ROS generation, resulting in oxidative macromolecule damage, can also contribute to cell proliferation leading to downstream skin tumor formation [7]. In addition, it has been shown that cell death accompanies cell proliferation during tumorigenesis, which may play both an eliminating and contributing role to carcinogenesis [13]. It has been suggested that MnSOD may be a novel tumor suppressor gene. We analyzed the ability of TPA to induce ROS generation in both clonal variants of JB6 cells and found that TPA induced a higher level of ROS generation in promotable JB6 P+ cells, which as mentioned above has lower MnSOD activity and higher levels of p53 expression and activation. Taken together, these results suggest a linkage between tumor promotion, mitochondrial ROS generation, p53-mediated apoptosis, and MnSOD activity.

MnSOD is a highly inducible protein, and when induced by dietary compounds such as Protandim, is effective in the suppression of tumor promotion [23]. The results from this study further confirmed and extended our previous findings that Protandim modulates tumorigenesis via the induction of endogenous antioxidant enzymes. In addition, Protandim utilizes multiple mechanisms to modulate cell proliferation and apoptosis *in vivo* and *in vitro*, which both contribute to tumorigenesis. Therefore, these results further demonstrate the effectiveness of multi-modal antioxidant base therapies in chemoprevention.

## Supporting Information

Figure S1 Ultrastructural detection of cutaneous apoptosis during early stage skin carcinogenesis. Mouse skin tissues were isolated from each treatment group. Ultrastructural features were used to identify apoptosis, such as cell shrinkage, chromatin condensation, dense nuclear staining, formation of cytoplasmic blebs and apoptotic bodies. Conventional electron microscopy, at low magnification, was used to obtain images of apoptotic and mitotic cells. The following ultrastructural features are labeled, apoptotic cells (Ap) were adjacent to mitotic cells (M) and areas of hyperplasia in the epidermal layer above the dermal layer (D), Bar  =  5 micrometer. The stratum corneum (SC) consists of keratin without cellular organelles.
(10.32 MB TIF)

Table S1 (0.06 MB DOC)

## References

[1] Zhao Y, Chaiswing L, Velez JM, Batinic-Haberle I, Colburn NH (2005). p53 translocation to mitochondria precedes its nuclear translocation and targets mitochondrial oxidative defense protein-manganese superoxide dismutase.. Cancer Res.

[2] Bowden GT, Finch J, Domann F, Krieg P (1995). Molecular mechanisms involved in skin tumor initiation, promotion, and progression..

[3] Avila GE, Zheng X, Cui XX, Ryan AD, Hansson A (2005). Inhibitory effects of 12-O-tetradecanoylphorbol-13-acetate alone or in combination with all-trans retinoic acid on the growth of cultured human pancreas cancer cells and pancreas tumor xenographs in immunodeficient mice.. J Pharmacol Exp Ther.

[4] Zheng X, Chang RL, Cui XX, Avila GE, Hebbar V (2006). Effects of 12-*O*-tetradecanoylphorbol-13-acetate (TPA) in combination with paclitaxel (Taxol) on prostate cancer LnCap cells cultured in vitro or grown as xenongraft tumors in immunodeficient mice.. Clin Cancer Res.

[5] Zhang X, Li W, Olumi AF (2007). Low dose 12-*O*-tetradecanoylphorbol-13-acetate enhances tumor necrosis factor related apoptosis-inducing ligand induced apoptosis in prostate cancer cells.. Clin Cancer Res.

[6] Polyak K, Xia Y, Zweier JL, Kinzler KW, Vogelstein B (1997). A model for p53-induced apoptosis.. Nature.

[7] Li P-F, Dietz R, von Harsdorf R (1999). p53 regulates mitochondrial membrane potential through reactive oxygen species and induces cytochrome *c*-independent apoptosis blocked by Bcl-2.. EMBO J.

[8] Tang X, Zhu Y, Han L, Kim AL, Kopelovich L (2007). CP-31398 restores mutant p53 tumor suppressor function and inhibits UVB-induced skin carcinogenesis in mice.. J Clin Invest.

[9] Liu J, Gu X, Robbins D, Li G, Shi R (2009). Protandim, a fundamentally new approach in chemoprevention using mouse two-stage skin carcinogenesis as a model.. PLoS One.

[10] Nelson SK, Bose SK, Grunwald GK, Myhill P, McCord JM (2006). The induction of human superoxide dismutase and catalase in vivo: a fundamentally new approach to antioxidant therapy.. Free Radic Biol Med.

[11] Manna SK, Mukhopadhyay A, Van NT, Aggarwal BB (1999). Silymarin suppresses TNF-induced Activation of NF-(kappa) B, cjun N-terminal kinase and apoptosis.. J Immunol.

[12] Buttke TM, Sandstorm PA (1994). Oxidative stress as a mediator of apoptosis.. Immunol Today.

[13] Hota SK, Barhwal K, Baitharu I, Prasad D, Singh SB (2009). Bacopa monniera leaf extract ameliorates hypobaric hypoxia induced spatial memory impairment.. Neurobiol Dis.

[14] Potapovich AI, Kostyuk VA (2003). Comparative study of antioxidant properties and cytoprotective activity of flavonoids.. Biochem (Mosc).

[15] Chaurasia SS, Panda S, Kar A (2000). Withania somnifera root extract in the regulation of lead-induced oxidative damage in male mouse.. Pharmacol Res.

[16] Cheng J, Tang XQ, Zhi JL, Cui Y, Yu HM (2006). Curcumin protects PC12 cells against 1-methyl-4-phenylpyridinium ion induced apoptosis by bcl-2-mitochondria-ROS- iNos pathway.. Apoptosis.

[17] Zhao Y, St Clair DK (2003). Detection of the content and activity of the transcription factor AP-1 in a multistage skin carcinogenesis model.. Methods Mol Biol.

[18] Zhao Y, Oberley TD, Chaiswing L, Lin S, Epstein CJ (2002). Manganese superoxide dismutase deficiency enhances cell turnover via tumor promoter-induced alterations in AP-1 and p53-mediated pathways in a skin cancer model.. Oncogene.

[19] Vaseva AV, Moll UM (2009). The mitochondrial p53 pathway.. Biochimica et Biophysica Acta.

[20] Katiyar SK, Roy AM, Baliga MS (2005). Silymarin induces apoptosis primarily through a p53-dependent pathway involving Bcl-2/Bax, cytochrome c release and caspase activation.. Mol Cancer Ther.

[21] Pani G, Koch OR, Galeotti T (2009). The p53-p66shc-manganese superoxide dismutase (MnSOD) network: A mitochondrial intrigue to generate reactive oxygen species.. The International J Biochem & Cell Biol.

[22] Pani G, Colavitti R, Bedogni B, Fusco S, Ferraro D (2004). Mitochondrial superoxide dismutase: a promising target for new anticancer therapies.. Curr Med Chem.

[23] Liu B, Chen Y, Clair DK St (2008). Ros and p53: a versatile partnership.. Free Radic Biol Med.

[24] Marchenko ND, Zaika A, Moll UM (2005). Death signal-induced localization of p53 protein to mitochondria. A potential role in apoptotic signaling.. J Biol Chem.

[25] Kroemer G, Reed JC (2000). Mitochondrial control of cell death.. Nat Med.

[26] Dhar SK, Xu Y, Chen Y, St. Clair DK (2006). Specificity Protein 1-dependent p53-mediated suppression of human manganese superoxide dismutase gene expression.. J Biol Chem.

