# Supplementary figures and images for: The Chemopreventive Effects of Protandim: Modulation of p53 Mitochondrial Translocation and Apoptosis during Skin Carcinogenesis
Source: PLoS One. 2010 Jul 30;5(7):e11902. doi: 10.1371/journal.pone.0011902 (PMC2912769; doi:10.1371/journal.pone.0011902)

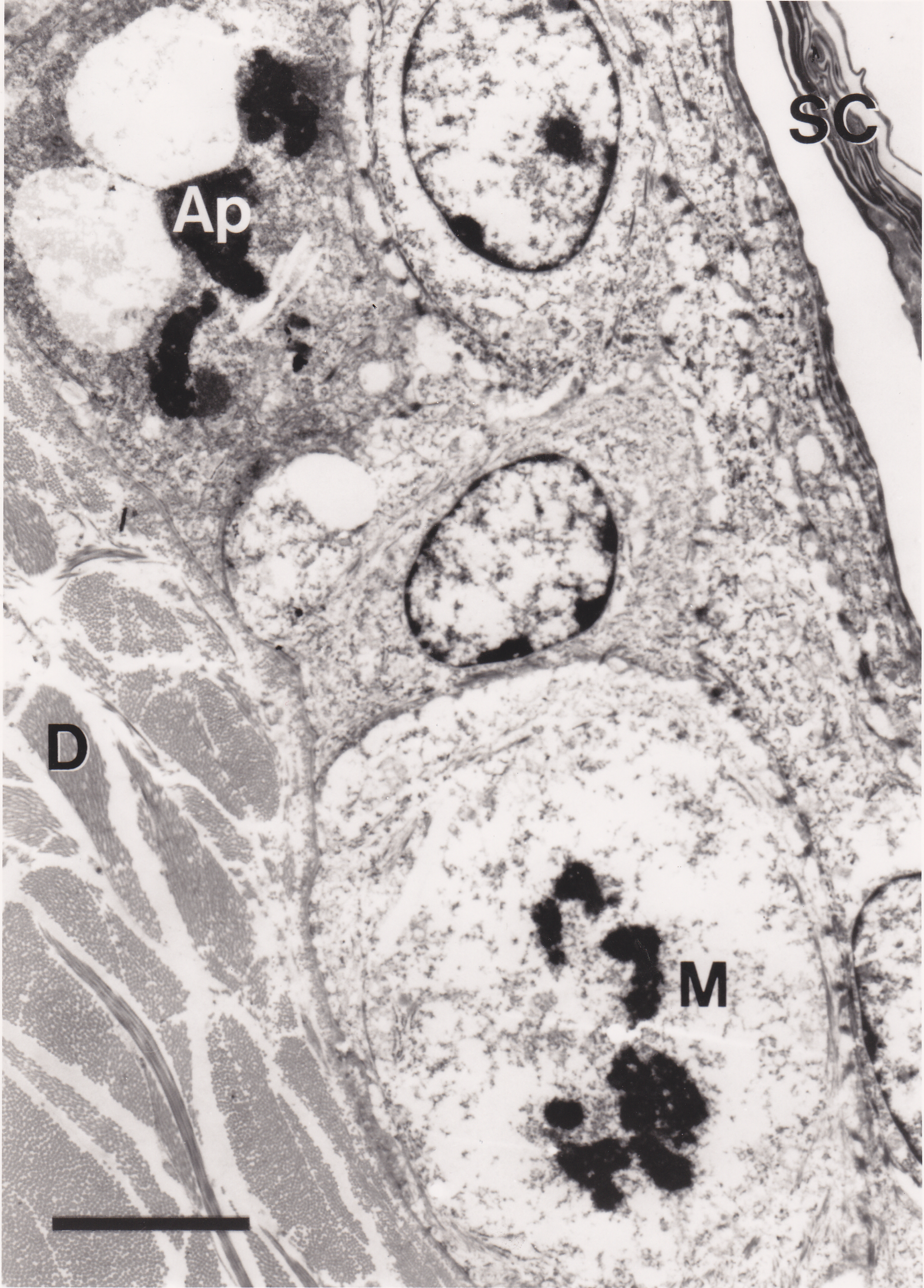

Supplement: Figure S1 — Ultrastructural detection of cutaneous apoptosis during early stage skin carcinogenesis. Mouse skin tissues were isolated from each treatment group. Ultrastructural features were used to identify apoptosis, such as cell shrinkage, chromatin condensation, dense nuclear staining, formation of cytoplasmic blebs and apoptotic bodies. Conventional electron microscopy, at low magnification, was used to obtain images of apoptotic and mitotic cells. The following ultrastructural features are labeled, apoptotic cells (Ap) were adjacent to mitotic cells (M) and areas of hyperplasia in the epidermal layer above the dermal layer (D), Bar = 5 micrometer. The stratum corneum (SC) consists of keratin without cellular organelles. (10.32 MB TIF) [file pone.0011902.s001.tif]
