# Supplementary material for: The Chemopreventive Effects of Protandim: Modulation of p53 Mitochondrial Translocation and Apoptosis during Skin Carcinogenesis
Source: PLoS One. 2010 Jul 30;5(7):e11902. doi: 10.1371/journal.pone.0011902 (PMC2912769; doi:10.1371/journal.pone.0011902)
Supplement: Supplementary file 2 [file pone.0011902.s002.doc]

Table S1. Colony formation in Soft Agar Transformation Assay.

**Treatment**	**Colonies formed/10^5 cells**
TPA 5	42.7 $\pm$ 3.2
Protandim/TPA	5.7 $\pm$ 1.8 *
Vehicle (EtOH)	4.3 $\pm$ 1.8
Protandim 1μl/ml	0 $\pm$ 0

p = 0.0006 compared with the TPA 5nM group.
